# Synthesis of Graphene Oxide-Coupled CoNi Bimetallic MOF Nanocomposites for the Simultaneous Analysis of Catechol and Hydroquinone

**DOI:** 10.3390/s23156957

**Published:** 2023-08-05

**Authors:** Shengbiao Zheng, Nini Zhang, Liang Li, Tianna Liu, Yuyang Zhang, Jing Tang, Jiahao Guo, Shao Su

**Affiliations:** 1 College of Chemistry and Material Engineering, Anhui Science and Technology University, Bengbu 233030, China; zhengsb@ahstu.edu.cn (S.Z.); 13586132394@163.com (N.Z.); ll18855614211@163.com (L.L.); 18119767585@163.com (T.L.); zhangyy@ahstu.edu.cn (Y.Z.); guojiahao1974@126.com (J.G.); 2 Anhui Province Quartz Sand Purification and Photovoltaic Glass Engineering, Research Center, Bengbu 233030, China; 3 State Key Laboratory of Organic Electronics and Information Displays & Jiangsu Key Laboratory for Biosensors, Institute of Advanced Materials (IAM), Nanjing University of Posts and Telecommunications, Nanjing 210023, China

**Keywords:** CoNi-MOF, graphene oxide, electrochemical, catechol, hydroquinone, simultaneous determination

## Abstract

Herein, a three-dimensional flower-like cobalt-nickel bimetallic metal-organic framework (CoNi-MOF) coupled with two-dimensional graphene oxide (GO) nanocomposites was successfully synthesized for the selective and simultaneous electrochemical determination of catechol (CC) and hydroquinone (HQ). The three-dimensional flower-like structure of the CoNi-MOF/GO nanocomposite has a multilayer structure and a large surface area, which greatly improves its electrocatalytic activity towards CC and HQ. Differential pulse voltammetry (DPV) results showed that the peak-to-peak separation of CC (0.223 V) and HQ (0.120 V) was 103 mV at a CoNi-MOF/GO modified glassy carbon electrode (CoNi-MOF/GO/GCE), suggesting that the proposed modified electrode can selectively and simultaneously determine them. Under optimal conditions, the CoNi-MOF/GO/GCE showed an excellent analytical performance for the simultaneous determination of CC and HQ, including a wide linear range (0.1–100 μM), low detection limit (0.04 μM for HQ and 0.03 μM for CC) and high anti-interference ability. As expected, the developed modified electrode has been used to analyze CC and HQ in river water, with acceptable results.

## 1. Introduction

Catechol (CC, *o*-dihydroxybenzene) and hydroquinone (HQ, *p*-dihydroxybenzene), two isomers of dihydroxybenzene, play a critical role in the food, pharmaceutical and chemical industries [1]. However, due to their strong irritation, high toxicity, high pollution and the difficulty of degrading them in the environment [2,3], they cause certain hazards to the ecosystem and human health. Moreover, it is difficult to selectively and simultaneously determine CC and HQ because their molecular structures, physical properties and chemical properties are very similar to each other. Therefore, several methods have been employed to selectively determine them, such as high performance liquid chromatography [4], chemiluminescence [5], fluorescence [6], electrochemistry [7,8,9,10], ultraviolet spectrophotometry [11] and so on. Among these detection methods, the electrochemical sensor has the features of an easy operation, low cost, high sensitivity and selectivity and has been considered an ideal tool for the rapid recognition and detection of CC and HQ [7,8,9,10].

The metal organic framework (MOF) is a novel type of porous material formed by the coordinated bonding of metal or metal cluster centers with organic ligands [12]. Recently, MOF has become an emerging sensing material due to its regular pore structure and large specific surface area [13,14,15]. However, its poor stability, low lattice vacancies and low electrical conductivity still hinder its electrochemical sensing application. To solve these problems, conductive materials and supporting substrates have been generally introduced into the construction of MOF-hybridized nanocomposites [16,17]. For example, Wang et al. introduced Co^2+^ ions to enhance the conductivity of Ni-based MOF nanocomposites. The doped Co can generate more “free holes” to promote the electrochemical performance of Co_2_-Ni-MOF nanocomposites [18]. Zhao et al. found that NiCo bimetal-organic framework nanosheets (NiCo-UMOFNs) had a high electrocatalytic activity towards the oxygen evolution reaction (OER) in alkaline conditions due to the introduction of Ni and Co metals [19]. Another solving approach involves coupling MOF with conductive materials, such as carbon nanotubes [20], graphene oxide [21], porous carbon [22,23], and MXene [24]. Among them, graphene oxide (GO) can efficiently increase the conductivity and stability of MOF-based electrocatalysts, which are particularly beneficial in the field of electroanalysis [21].

**Figure 1:**
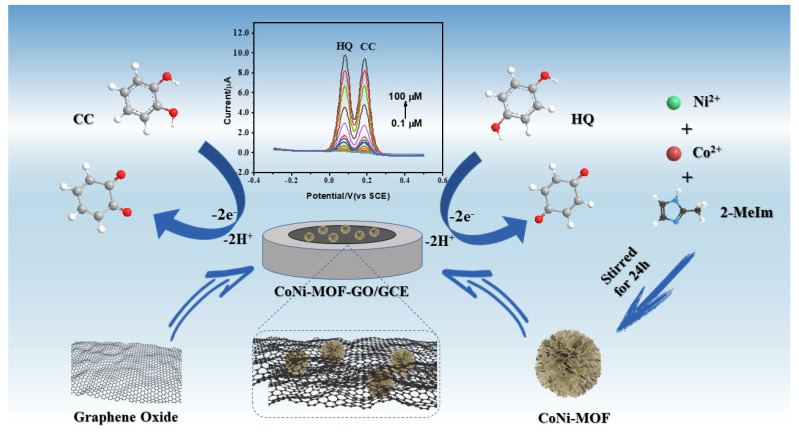
The fabrication of CoNi-MOF/GO/GCE and its application in the electrochemical determination of catechol and hydroquinone. The arrows mean oxidation process on the electrode interface.

Inspired by the above considerations, a nanocomposite consisting of graphene oxide and a CoNi bimetallic MOF was synthesized. The preparation process of CoNi-MOF/GO and electrochemical detection mechanism of this electrochemical sensor are illustrated in Figure 1. In the initial step of the reaction process, Ni^2+^ and Co^2+^ could be easily adsorbed onto the surface of GO due to a large number of carboxyl and hydroxyl groups located on the surface of GO, resulting in the constitution of CoNi-MOF on the surface of GO. On the basis of the successful synthesis, the CoNi-MOF/GO nanocomposite was used as the electrode-modified material to construct a high-performance electrochemical sensor for the selective and simultaneous determination of CC and HQ. Due to the synergistic catalytic effect of the CoNi-MOF/GO nanocomposite, the as-prepared electrochemical sensor exhibits an excellent sensitivity, selectivity and stability, which can be used to determine CC and HQ in actual river water.

## 2. Experimental Section

### 2.1. Reagents, Chemicals and Characterization

The reagents, chemicals and characterization equipment are listed in Supporting Information.

### 2.2. Synthesis of CoNi-MOF

The bimetallic MOF nanoflowers were synthesized based on the literature [25]. A total of 12 mmol Co(NO_3_)_2_·6H_2_O and 4 mmol Ni(NO_3_)_2_·6H_2_O were co-dissolved in methanol. A total of 4 mmol 2-methylimidazole was dissolved in methanol. Then, the two solutions were rapidly mixed and stirred for 2 min. Subsequently, the mixed solution was left for 24 h for the growth and aging of MOF crystals. After growth, the mixture was centrifuged and washed with methanol three times. Finally, the product was dried in a vacuum at 60 °C for 12 h to obtain an earthy yellow MOF powder.

### 2.3. Synthesis of Graphene Oxide (GO)

Graphene oxide was obtained based on Hummers’ method [26]. A concentrated mixture of 9:1 H_2_SO_4_/H_3_PO_4_ was added to graphite flakes. Then, the mixture was heated to 50 °C and stirred for 12 h. After reaction, the mixture was poured into ice with 30% H_2_O_2_ to obtain aqueous solvent GO. Subsequently, the obtained GO was washed and purified by H_2_O, 30% HCl and ethanol, respectively. Finally, GO was dispersed in ethanol for storage.

### 2.4. Fabrication of the CoNi-MOF/GO

For the synthesis of CoNi-MOF/GO, 12 mmol Co(NO_3_)_2_·6H_2_O and 4 mmol Ni(NO_3_)_2_·6H_2_O were firstly dissolved together in methanol. Then, 1.5 mL GO solution was added to the above solution and sonicated for 30 min until complete dispersion. After that, 4 mmol 2-methylimidazole was added in the above suspension and stirred for 24 h. Subsequently, the mixture was centrifuged and washed four times with methanol. Finally, the product was lyophilized in a freeze dryer for 22 h.

### 2.5. Construction of the Modified Electrode

A total of 1 mg/mL CoNi-MOF/GO suspension was modified on the surface of the pretreated bare glassy carbon electrode to obtain the expected modified electrode, which was defined as CoNi-MOF/GO/GCE. Other modified electrodes were prepared with a similar procedure.

## 3. Results and Discussion

### 3.1. Characterization of CoNi-MOF/GO Nanocomposites

**Figure 2:**
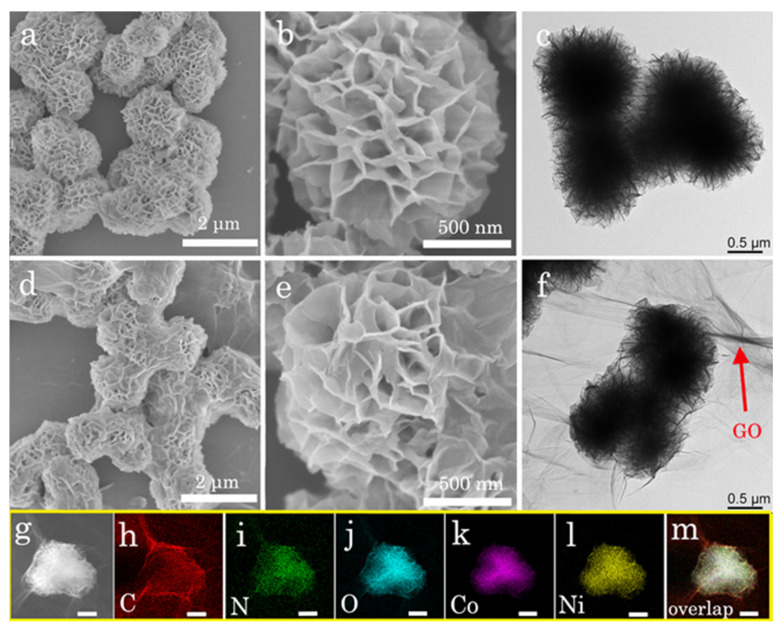
Characterization of CoNi-MOF and CoNi-MOF/GO nanocomposites. SEM images of (**a**) CoNi-MOF and (**d**) CoNi-MOF/GO nanocomposites at low magnification (scale bar: 2 μm). SEM images of (**b**) CoNi-MOF and (**e**) CoNi-MOF/GO nanocomposites at high magnification (scale bar: 500 nm). TEM images of (**c**) CoNi-MOF and (**f**) CoNi-MOF/GO nanocomposites. (**g**) HAADF-STEM of CoNi-MOF/GO nanocomposites. (**h**–**m**) The corresponding EDS mapping images of C, N, O, Co and Ni elements and the overlapped mapping images (scale bar: 1 μm).

The morphologies of CoNi-MOF and CoNi-MOF/GO nanocomposites were recorded by transmission electron microscopy (TEM) and scanning electron microscopy (SEM). From Figure 2a,b, CoNi-MOF shows a typical porous flower-like structure with many channels. With the introduction of GO, CoNi-MOF easily binds with GO to form CoNi-MOF/GO nanocomposites (Figure 2d,e). One can note that the morphologies of CoNi-MOF are still porous flower-like structures. TEM images were agreement with the SEM results, proving the successful synthesis of CoNi-MOF/GO nanocomposites (Figure 2c,f). EDS results show that C, N, O, Ni and Co elements are uniformly distributed in the CoNi-MOF/GO nanocomposite, further confirming the successful combination of CoNi-MOF and GO (Figure 2g–m). The hierarchical structure of CoNi-MOF/GO has a large number of active sites, which can facilitate electron transfer on its interface in the redox process [27].

**Figure 3:**
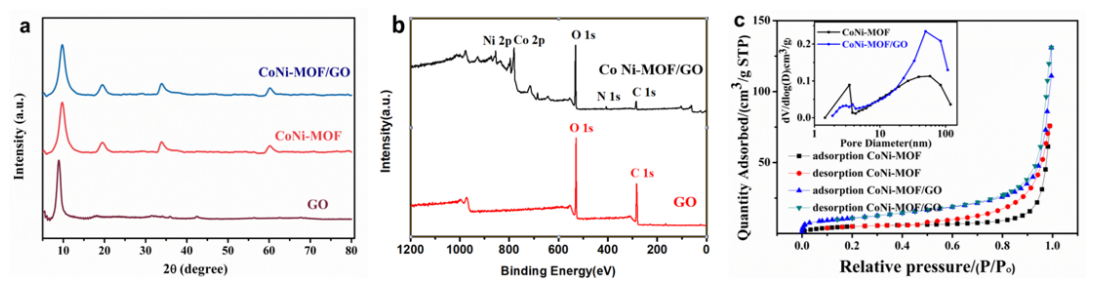
(**a**) XRD spectra of the CoNi-MOF/GO. (**b**) XPS survey spectrum of CoNi-MOF/GO and GO. (**c**) N_2_ adsorption–desorption isotherms of CoNi-MOF and CoNi-MOF/GO. Inset: Barrett–Joyner–Halenda pore size distribution.

The structures of CoNi-MOF, CoNi-MOF/GO and GO were interpreted by XRD (Figure 3a). A distinct diffraction peak belonged to the (001) crystal planes of the GO spectrum, which was consistent with the previous characteristics of GO [28]. Additionally, the diffraction peaks of CoNi-MOF were located at 9.88°, 19.36°, 33.81°, and 60.47°, which is in agreement with previous observations of the Ni-Co metal organic skeleton [29]. One can note that all patterns of CoNi-MOF and CoNi-MOF/GO are remarkably similar due to the overlap of the diffraction peak around 9°. The reason is ascribed to GO being used as a supporting substrate to load CoNi-MOF. As a result, the introduction of GO did not disrupt the crystallinity of CoNi-MOF. The elemental composition of CoNi-MOF/GO nanocomposites was investigated by X-ray photoelectron spectroscopy (XPS). As shown in Figure 3b, the typical XPS survey spectra of Ni 2p, Co 2p and N 1s were observed in CoNi-MOF/GO nanocomposites compared with GO, suggesting the successful synthesis of bimetal CoNi-MOF. Notably, this result is in good agreement with the EDX results, suggesting the successful synthesis of CoNi-MOF/GO nanocomposites.

In addition, the specific surface areas of CoNi-MOF and CoNi-MOF/GO nanocomposite were studied. According to the nitrogen adsorption and desorption plots (Figure 3c), the specific surface area of CoNi-MOF/GO nanocomposites (39.3605 m^2^·g^−1^) is larger than that of CoNi-MOF (20.4520 m^2^·g^−1^). Both CoNi-MOF and CoNi-MOF/GO samples show typical mesoporous structures with pore size distributions in the range of 20.0 to 60.0 nm (the inset of Figure 3c). Moreover, GO does not affect the pore size distributions of CoNi-MOF. CoNi-MOF/GO nanocomposites have large specific surface areas and mesoporous structures, which is favorable for the adsorption of CC and HQ and is beneficial to improving the electrochemical performance.

### 3.2. Electrochemical Behaviors of CoNi-MOF/GO/GCE

**Figure 4:**
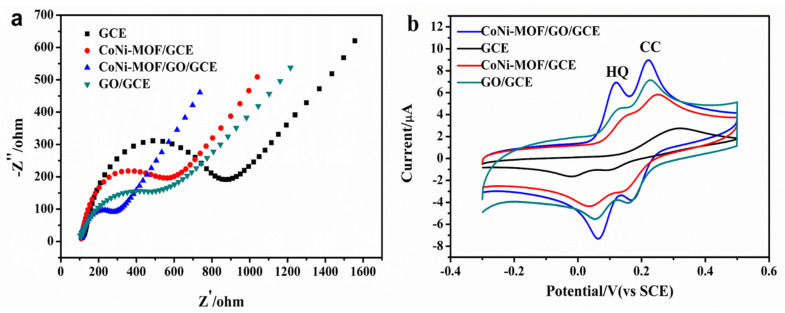
(**a**) EIS spectra of bare GCE, CoNi-MOF/GO/GCE, GO/GCE and CoNi-MOF/GCE. (**b**) CVs of 1 × 10^−4^ M CC and HQ in phosphate buffer (pH 6.0) at bare GCE, CoNi-MOF/GCE, GO/GCE and CoNi-MOF/GO/GCE with a scan rate of 100 mV·s^−1^.

The electrochemical behaviors of CoNi-MOF/GO/GCE were tested by electrochemical impedance spectroscopy (EIS) and cyclic voltammetry (CV). The impedance variations of different electrodes in [Fe(CN)_6_]^3−/4−^ solution are shown in Figure 4a. The impedance value of Co-Ni-MOF/GCE (480 Ω) is smaller than that of bare GCE (738 Ω), suggesting that Co-Ni-MOF improves the electron transfer to some extent. Meanwhile, the impedance value of GO/GCE (450 Ω) is smaller than that of Co-Ni-MOF/GCE because of the good electrical conductivity of GO. The impedance value of CoNi-MOF/GO/GCE is down to 186 Ω, proving that the CoNi-MOF/GO nanocomposites have a better electrical conductivity. The introduction of GO greatly improves the conductivity due to the synergistic effect of CoNi-MOF and GO, which offers a possibility for the electrochemical determination of CC and HQ.

According to their excellent conductivity, the electrochemical responses of different modified electrodes for the determination of CC and HQ were recorded in Figure 4b. For the 1.0 × 10^−4^ mol/L CC and HQ determination in phosphate buffer (pH 6.0), only a board oxidation peak at 319 mV was observed at bare GCE, with a low oxidation peak current (*I*_pa_ = 1.821 μA). Obviously, bare GCE cannot efficiently distinguish CC from HQ. At the same detection condition, two pairs of well-defined redox peaks for HQ and CC were found at GO/GCE, with an oxidation peak-to-peak separation of 101 mV, indicating that GO/GCE had a better electrocatalytic activity and could efficiently determine two isomers. Two pairs of redox peaks located at 235 mV/156 mV for CC and 138 mV/53 mV for HQ were also obtained at CoNi-MOF/GCE. The oxidation peak potential shifted negatively, indicating that CoNi-MOF/GCE can lower the oxidation overpotential of CC and HQ. The low oxidation is beneficial for the electrochemical determination of CC and HQ. At CoNi-MOF/GO/GCE, two well-defined oxidations peaks of CC and HQ appeared at 0.223 V and 0.120 V, respectively, further lowering the overpotential. The oxidation peak–peak separation between CC and HQ is about 103 mV, suggesting that it is enough for the simultaneous determination of CC and HQ. Moreover, the oxidation peak currents of CC and HQ at CoNi-MOF/GO/GCE are about 4.897 μA and 5.359 μA, respectively, which is almost twice those obtained at CoNi-MOF/GCE or GO/GCE. This significant enhancement may be related to the combination of CoNi-MOF with GO. The as-prepared CoNi-MOF/GO nanocomposites have an excellent electrical conductivity, which is favorable for the improvement of the electrochemical response of CC and HQ on the electrode surface.

### 3.3. Effect of pH Value and Scan Rate

**Figure 5:**
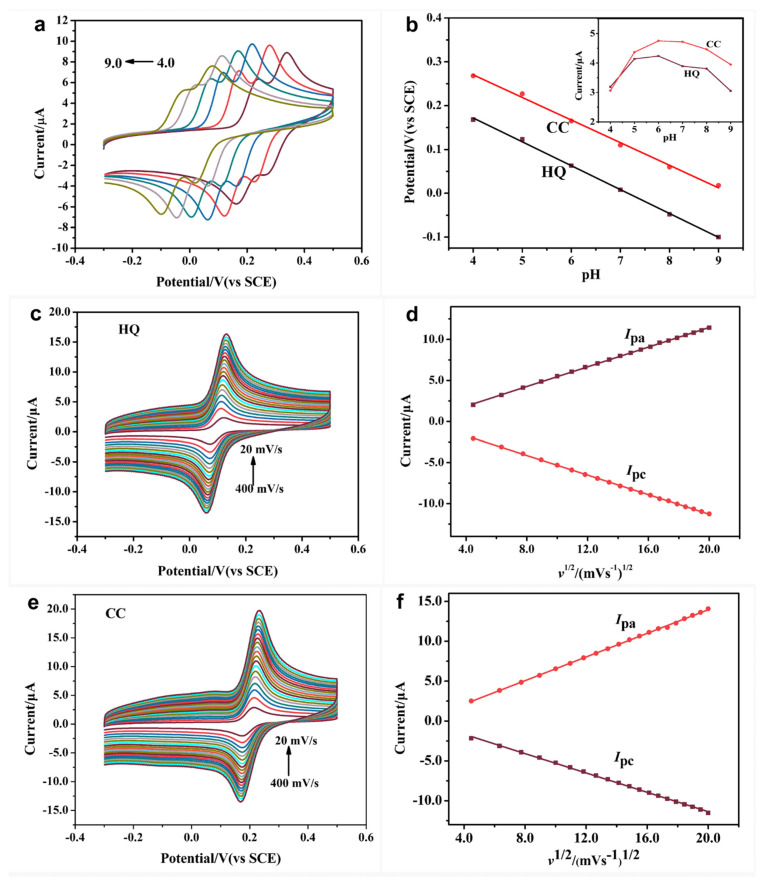
(**a**) CVs of 1 × 10^−4^ M CC and HQ at CoNi-MOF/GO/GCE in phosphate buffer with different pH (4.0–9.0). (**b**) pH value versus peak potential of CC and HQ. Inset: Relationship between pH value and peak current of CoNi-MOF/GO/GCE for CC and HQ determination. CV curves of 1 × 10^−4^ M (**c**) HQ and (**e**) CC at CoNi-MOF/GO/GCE at different scan rates (20~400 mV·s^−1^), and (**d**,**f**) plots of *I*_pa_/*I*_pc_ of CC and HQ versus the square root of the scan rate.

The effects of the pH value and scan rate on the electrochemical behavior of CC and HQ at CoNi-MOF/GO/GCE were studied. As shown in Figure 5a, the peak potentials of CC and HQ shifted negatively with the increasing pH value ranging from 4.0 to 9.0, suggesting that protons participated in the redox process [30,31] (Figure 5b). The relationship between the pH value and the anodic peak potentials (*E*_pa_) of CC and HQ was expressed as follows:

*E*_pa(HQ)_ = 0.3912 − 0.0545 pH (R^2^ = 0.9988), *E*_pa(CC)_ = 0.4767 − 0.0516 pH (R^2^ = 0.9967)

The slopes of both linear equations are close to the Nernst equation (0.059 V/pH), proving that an equal number of electrons and protons are involved in the oxidation processes of CC and HQ. The corresponding oxidation mechanisms of CC and HQ at CoNi-MOF/GO/GCE are given in Figure 1, which is consistent with the previous literature [10,32]. Meanwhile, the largest anodic peak currents of CC and HQ were obtained when the pH value was 6.0. For the best detection performance, pH 6.0 is chosen as the optimal pH value for the electrochemical determination (inset in Figure 5b).

For the purpose of researching the electrochemical reaction kinetics of CC and HQ at CoNi-MOF/GO/GCE, the CV responses were performed by changing the scan rates from 20 to 400 mV/s (Figure 5c,e). From Figure 5d,f, it can be seen that the peak currents of CC and HQ increased with the increase of the scan rate. Both redox peak currents of CC and HQ are proportional to the square root of the scan rate. The fitting equations were listed as follows:

CC: *I*_pa_ (μA) = 0.6068 *v*^1/2^ − 0.7667 (R^2^ = 0.9992), *I*_pc_ (μA) = −0.7413 *v*^1/2^ +0.8681 (R^2^ = 0.9995)

HQ: *I*_pa_ (μA) = 0.5814 *v*^1/2^ − 0.3879 (R^2^ = 0.9963), *I*_pc_ (μA) = −0.6005 *v*^1/2^ + 0.5456 (R**^2^** = 0.9997)

According to the experimental data, the electrochemical catalyses of CC and HQ at CoNi-MOF/GO/GCE are diffusion-controlled processes [33].

### 3.4. Selective and Simultaneous Detection of CC and HQ

**Figure 6:**
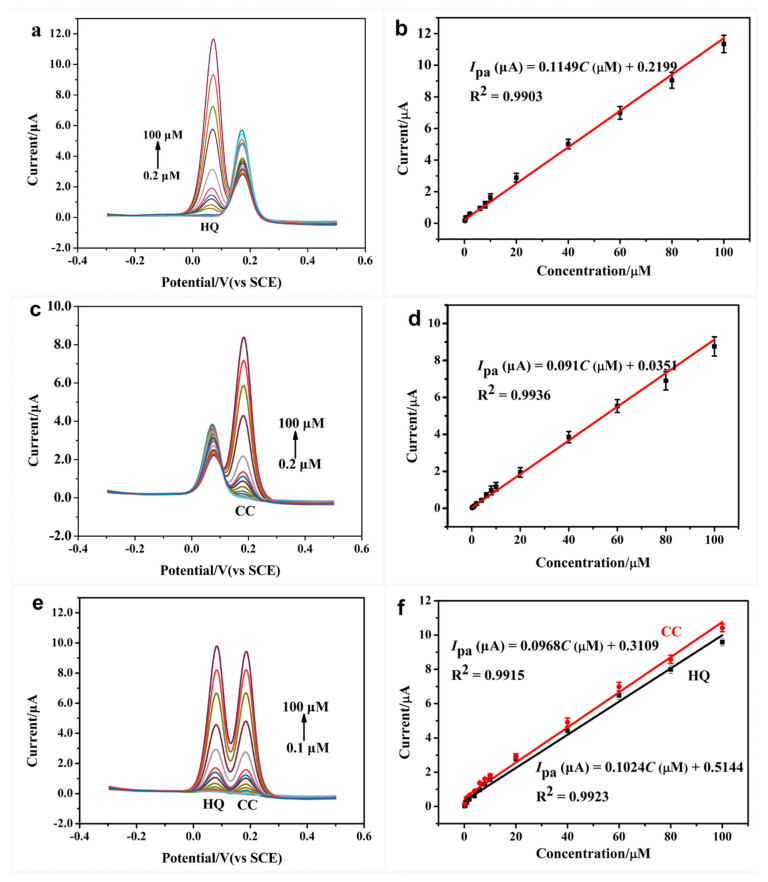
(**a**) DPV curves of CoNi-MOF/GO/GCE for 0.2~100 μM HQ determination in the presence of 20 μM CC. (**b**) The corresponding linear range between oxidation peak current and HQ concentration. (**c**) DPV curves of CoNi-MOF/GO/GCE for 0.2~100 μM CC by fixing HQ concentration (20 μM). (**d**) The relationship between the peak current and the CC concentration. (**e**) DPV curves of CoNi-MOF/GO/GCE for simultaneous determination of 0.1~100 μM CC and 0.1~100 μM HQ. (**f**) Calibration plots of the oxidation peak currents as a function of CC and HQ concentration, respectively.

Differential pulse voltammetry (DPV) was used to evaluate the performance of CoNi-MOF/GO/GCE for the determination of CC and HQ. For the selective determination, one substance concentration is changing when the other substance concentration is fixed. Figure 6a shows that the anodic peak currents of HQ increase proportionally to the increasing HQ concentration in the range of 0.2–100 μM, while the peak currents of 20 μM CC remain unchanged. The linear regression equation of CoNi-MOF/GO/GCE for HQ detection was *I_pa_* (μA) = 0.1149 C (μM) + 0.2199 (R^2^ = 0.9903), and the limit of detection (LOD) was estimated to be 0.005 μM (Figure 6b, S/N = 3). A similar phenomenon was obtained for the CC determination at CoNi-MOF/GO/GCE (Figure 6c). The oxidation peak currents of CC were linear with a CC concentration ranging from 0.2 to 100 μM, while the HQ concentration (20 μM) remained constant. The fitting equation for CC detection is *I*_pa_ (μA) = 0.091 C (μM) + 0.0351 (R^2^ = 0.9936) with a LOD of 0.004 μM (Figure 6d, S/N = 3).

Besides the selective determination, the simultaneous analysis of CC and HQ at CoNi-MOF/GO/GCE was also investigated by changing their concentration synchronously. As shown in the result represented in Figure 6e, two well-separated anodic peaks for HQ and CC are exhibited. The anodic peak currents of CC and HQ increased with the addition of two isomer concentrations ranging from 0.1 to 100 μM, respectively. The obtained linear equations for the CC and HQ determination were *I*_pa_ (μA) = 0.0968 C (μM) + 0.3109 (R^2^ = 0.9915) and *I*_pa_ (μA) = 0.1024 C (μM) + 0.5144 (R^2^ = 0.9923), respectively (Figure 6f). According to regression equations, the LODs for HQ and CC detection were calculated to be 0.04 μM and 0.03 μM, respectively. All experimental data suggested that CoNi-MOF/GO/GCE can selectively and simultaneously determine CC and HQ without them interfering with each other.

The analytical performance of CoNi-MOF/GO/GCE for the CC and HQ determination is better or comparable to some published modified electrodes (Table 1), suggesting that the outstanding synergistic activity of CoNi-MOF/GO can efficiently improve electrochemical detection performance.

### 3.5. Reproducibility, Stability and Selectivity

Six independent CoNi-MOF/GO/GCEs were used to detect 20 μM CC and 20 μM HQ. The relative standard deviations (RSDs) of the anodic peak currents were found to be 1.79% and 2.04% for CC and HQ, respectively (Figure S1). After eight consecutive determinations for the same sensor, the RSDs of anodic peak currents were about 2.83% and 1.97% for the CC and HQ determination, respectively (Figure S2). These results indicate that CoNi-MOF/GO/GCE has a satisfactory reproducibility and repeatability. After the CoNi-MOF/GO/GCE was stored at room temperature for three weeks, the anodic peak currents of CC and HQ still remained above 90% of the original current (Figure S3), indicating that CoNi-MOF/GO/GCE possesses an excellent long-storage stability.

**Figure 7:**
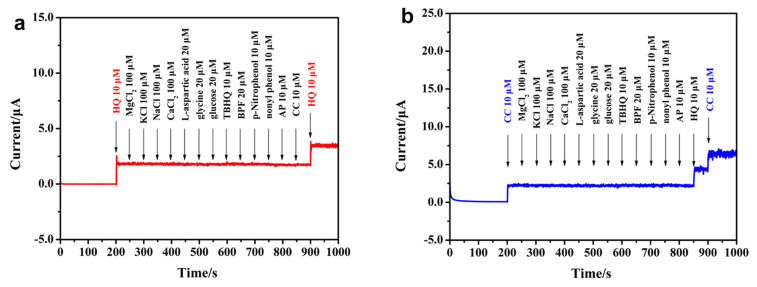
I-t curves of CoNi-MOF/GO/GCE for (**a**) HQ and (**b**) CC detection in the presence of different interfering substances.

The anti-interference ability of CoNi-MOF/GO/GCE for the CC and HQ determination was also verified. Figure 7a exhibits the amperometric responses of CoNi-MOF/GO/GCE for HQ (10 μM) and other interfering substances’ (100 μM of Na^+^, K^+^, Mg^2+^ and Ca^2+^, 20 μM of L-aspartic acid, glycine, BPF, tertbutyl-hydroquinone and glucose, 10 μM of p-nitrophenol, nonylphenol and acetaminophen) determination. The applied potential is 0.14 V. Obviously, other interfering substances did not affect the performance of CoNi-MOF/GO/GCE for HQ determination. A similar phenomenon was observed for CC detection at CoNi-MOF/GO/GCE (Figure 7b), proving that these co-existing substances did not significantly interfere in the detection of CC. All experimental data suggested that CoNi-MOF/GO/GCE has a good selectivity for CC and HQ determination.

The reliability of CoNi-MOF/GO/GCE was investigated. A standard addition method was employed to determine the content of CC and HQ in Huaihe River water. The preparation of Huaihe River samples is listed in the Supporting Information. As tabulated in Table 2, the recoveries of CoNi-MOF/GO/GCE for the HQ and CC determination ranged from 98.1–103.7% (RSD < 3%, *n* = 3) and 96.9–102.9% (RSD < 4%, *n* = 3). Therefore, the fabricated CoNi-MOF/GO/GCE has a good practicality for the detection of CC and HQ, proving that it has a great application in real sample analysis.

## 4. Conclusions

In conclusion, a hybridized nanocomposite combining cobalt-nickel bimetallic MOFs with graphene oxide was synthesized and was used as an electrode modifier to construct an electrochemical sensor for the selective and simultaneous determination of CC and HQ. Owing to the high catalytic activity, good conductivity and specific surface area of CoNi-MOF/GO nanocomposites, the proposed sensor is endowed with an excellent analytical performance for CC and HQ, such as a wide linear range, excellent selectivity and good stability. This work provides a feasible model for constructing simple, high-performance bimetallic MOF-based electrochemical sensors in environmental monitoring.

## Figures and Tables

**Table 1:** Comparison of different modified electrodes for simultaneous analysis of CC and HQ.

Materials	Linear Range (μM)		Detection Limit (μM)		References
	HQ	CC	HQ	CC	
Ce-MOF/CNTs/GCE	10–100	5–50	5.3	3.5	[8]
RGO-MWNTs	8–391	5.5–540	2.6	1.8	[9]
MCHSs/Co@N-CNTs/GCE	1.0–100	2.5–100	0.27	0.46	[12]
AuPdNF/rGO/GCE	1.6–100	2.5–100	0.5	0.8	[2]
Co_3_O_4_@carbon/GCE	0.8–127.1	0.6–116.4	0.03	0.03	[34]
CuS-CNF/GCE	3–200	7–150	0.293	0.259	[35]
COFs/MWCNT/GCE	4–450	4–450	0.38	0.36	[36]
UiO-66-NH_2_/COCl-MWCNT/CB/GCE	1–1000	1–1000	0.27	0.11	[37]
CoNi-MOF/GO/GCE	0.1–100	0.1–100	0.03	0.04	This work

**Table 2:** Determination of CC and HQ in Huaihe River samples by using CoNi-MOF/GO/GCE (*n* = 3).

Sample	Analyte	Added (μM)	Found ^a^ (μM)	RSD ^b^ (%)	Recovery (%)
1	HQ	20	20.33 ± 0.42	2.06	111.7
	CC	20	19.46 ± 0.68	3.50	97.4
2	HQ	40	39.24 ± 0.94	2.40	98.1
	CC	40	38.75 ± 0.82	2.12	96.9
3	HQ	50	51.83 ± 1.48	2.85	103.7
	CC	50	51.45 ± 1.27	2.47	102.9

^a^ Standard addition method. ^b^ Measurement values taken from three experiments.

## Data Availability

Data can be available on request.

## Supplementary Materials

The following supporting information can be downloaded at: https://www.mdpi.com/article/10.3390/s23156957/s1, Figure S1: Reproducibility study of six individual electrodes for CC and HQ detection under the same conditions; Figure S2: The same electrode for 8 consecutive determinations under the same conditions; Figure S3: Stability of CoNi-MOF/GO/GCE.
