# Supplementary material for: Synthesis of Graphene Oxide-Coupled CoNi Bimetallic MOF Nanocomposites for the Simultaneous Analysis of Catechol and Hydroquinone
Source: Sensors (Basel). 2023 Aug 5;23(15):6957. doi: 10.3390/s23156957 (PMC10422656; doi:10.3390/s23156957)
Supplement: Supplementary file 1 [file sensors-23-06957-s001.zip › sensors-2487730-supplementary.pdf]

Supporting information

Synthesis of Graphene Oxide-Coupled CoNi Bimetallic MOF Nanocomposites for the Simultaneous Analysis of Catechol and Hydroquinone

Shengbiao Zheng [1,2], Nini Zhang [1], Liang Li [1], Tianna Liu [1], Yuyang Zhang [1,2], Jing Tang [1,2,*], Jiahao Guo [1,2] and Shao Su [3,*]

[1] College of Chemistry and Material Engineering, Anhui Science and Technology University, Bengbu 233030, China; zhengsb@ahstu.edu.cn (S.Z.); 13586132394@163.com (N.Z.); ll18855614211@163.com (L.L.); 18119767585@163.com (T.L.); zhangyy@ahstu.edu.cn (Y.Z.); guojiahao1974@126.com (J.G.)

[2] Anhui Province Quartz Sand Purification and Photovoltaic Glass Engineering, Research Center, Bengbu 233030, China

[3] State Key Laboratory of Organic Electronics and Information Displays & Jiangsu Key Laboratory for Biosensors, Institute of Advanced Materials (IAM), Nanjing University of Posts and Telecommunications, Nanjing 210023, China

* Correspondence: zhengtang102@163.com (J.T.); iamssu@njupt.edu.cn (S.S.)

Reagents and Chemicals

Catechol, hydroquinone, cobalt nitrate hexahydrate; nickel nitrate hexahydrate, 2-methylimidazole, p-nitrophenol, nonylphenol, 4-acetylaminophenol, methanol, ethanol, L-aspartic acid, glycine, BPF, tertbutyl-hydroquinone and glucose were produced by Aladdin Industries (China). Sodium nitrate, N, N-dimethylformamide, graphene, sulfuric acid, NaH_2PO_4, Na_2HPO_4, HCl, H_2O_2, potassium nitrate, magnesium chloride and calcium chloride were produced by Sinopharm Chemical Reagent Co. The phosphate buffer solution used was a mixture of NaH_2PO_4 (0.1 M) and Na_2HPO_4 (0.1 M). All reagents were not further purified.

Characterization

The morphology of the composites was analyzed by scanning electron microscopy (SEM, JSM-7100F) and transmission electron microscopy (TEM, JEM-100CX JEOL), respectively. Powder x-ray diffraction (XRD) was acquired with a Bruker D8-ADVANCE with Cu Kα radiation. N_2 adsorption/desorption isotherms were conducted on a NOVA Touch LX2 analyzer (Quantachrome) at 77 K. The surface chemical compositions were performed on a K-Alpha X-ray photoelectron spectroscopy (XPS) (Thermo Fisher Scientific). EIS, CV, DPV were performed at a CHI 660E electrochemical instrument (Shanghai Chenhua), where a three-electrode cell with different modified glassy carbon electrodes, saturated calomel electrode (SCE) and platinum wire were used as working, auxiliary and reference electrode, respectively.

Preparation of River samples

At first, a 20 mL sample of Huaihe River water was centrifuged at 3000 rpm for 15 min. Then, this water was filtered twice by using filter paper to remove impurities. Finally, the purified Huaihe River was dissolved in 200 mL phosphate buffer (pH = 6.0) for dilution. For the quantitative analysis of CC and HQ in Huaihe River water, we added known amounts of CC and HQ (20, 40, 50 μM) in above Huaihe River sample by using standard addition method (n=3). According to the equation, the recoveries of this sensor were calculated.

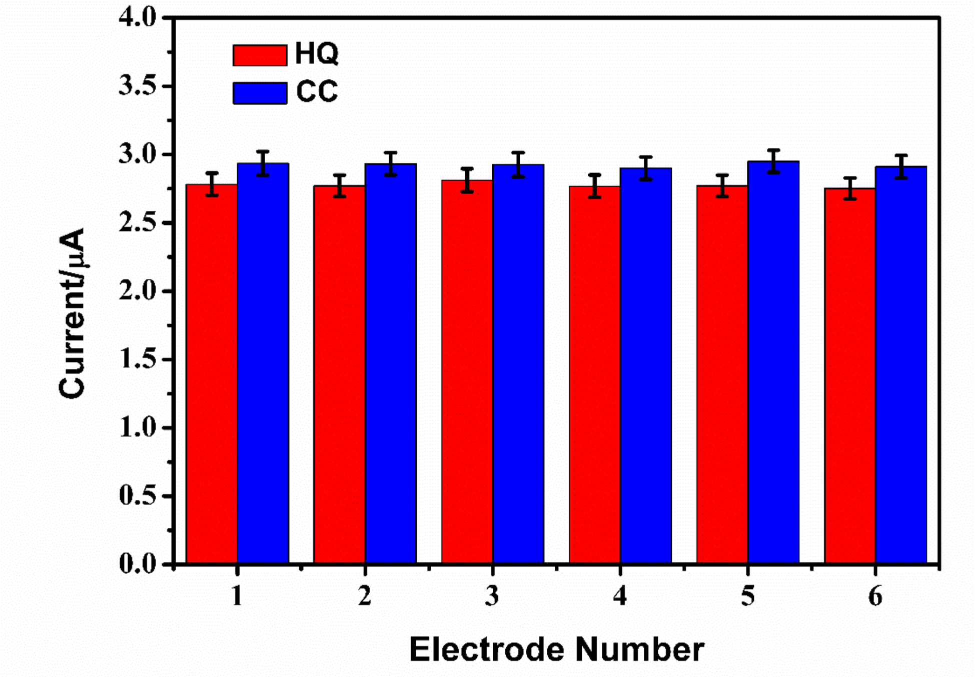

Figure S1. Reproducibility study of six individual electrodes for CC and HQ detection under the same conditions.

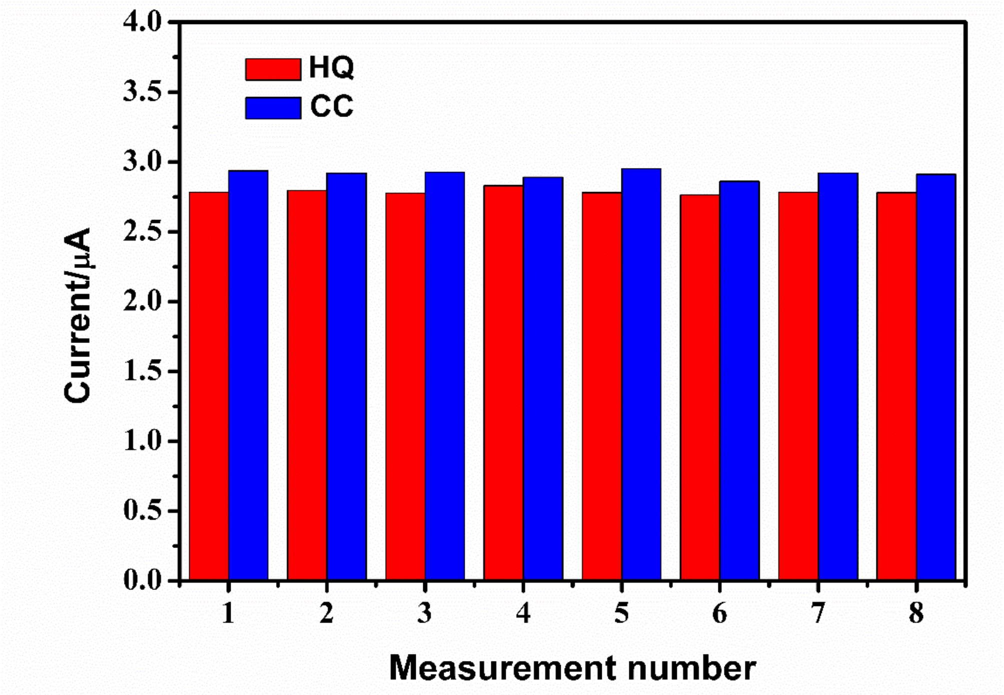

Figure S2. The same electrode for 8 consecutive determinations under the same conditions.

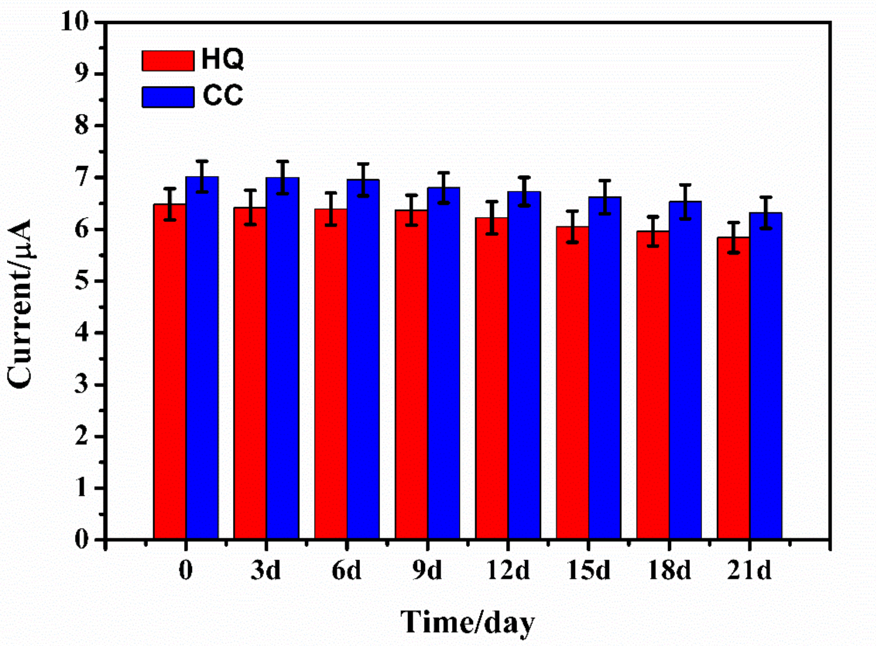

Figure S3. Stability of CoNi-MOF/GO/GCE.
